# Weighted Mean Squared Deviation Feature Screening for Binary Features

**DOI:** 10.3390/e22030335

**Published:** 2020-03-14

**Authors:** Gaizhen Wang, Guoyu Guan

**Affiliations:** 1 School of Mathematics and Statistics, Northeast Normal University, Changchun 130000, China; wanggz828@nenu.edu.cn; 2 Key Laboratory for Applied Statistics of the MOE, School of Economics and Management, Northeast Normal University, Changchun 130000, China

**Keywords:** Chi-square statistic, feature screening, mutual information, Pearson correlation coefficient, power-law distribution, weighted mean squared deviation

## Abstract

In this study, we propose a novel model-free feature screening method for ultrahigh dimensional binary features of binary classification, called weighted mean squared deviation (WMSD). Compared to Chi-square statistic and mutual information, WMSD provides more opportunities to the binary features with probabilities near 0.5. In addition, the asymptotic properties of the proposed method are theoretically investigated under the assumption logp=o(n). The number of features is practically selected by a Pearson correlation coefficient method according to the property of power-law distribution. Lastly, an empirical study of Chinese text classification illustrates that the proposed method performs well when the dimension of selected features is relatively small.

## 1. Introduction

Feature screening is a practical and powerful tool in data analysis and statistical modeling of ultrahigh dimensional data, such as genomes, biomedical images and text data. In supervised learning, features of data often satisfy the sparsity assumption, i.e., only a small number of features are relevant to the response in a large amount of features. Therefore, Fan and Lv [1] proposed a sure independence screening method based on correlation learning for linear model and theoretically proved the screening consistency. Subsequently, a series of model-free feature screening methods were proposed, which did not require model specification [2,3,4,5,6,7]. These methods learned the marginal relationships between the response and features, and filtered out the features with weak relationships to response.

In this study, we focus on feature screening of binary classification with ultrahigh dimensional binary features. The purpose of feature screening in classification is to filter out a large amount of irrelevant features that are unhelpful for the discrimination of class labels. Both computational speed and classification accuracy are also expected to be taken into account. For categorical features, statistical test (e.g., Chi-square test) [8,9], information theory (e.g., information gain, mutual information, cross entropy) [10,11,12,13], and Bayesian methods [14,15] are usually used for feature screening, especially in the field of text classification. In this study, we propose a novel model-free feature screening method called weighted mean squared deviation (WMSD), which can be considered as a simplified version of Chi-square statistic and mutual information. Next, according to the property of power-law distribution [16,17], a Pearson correlation coefficient method is developed to select the number of the relevant features. Lastly, the proposed method is applied to Chinese text classification. It outperforms Chi-square statistic and mutual information when a small number of words are selected.

The rest of this article is organized as follows. In Section 2.1, we introduce the weighted mean squared deviation feature screening method and investigate its asymptotic properties. In Section 2.2, a Pearson correlation coefficient method is developed based on the property of power-law distribution for model selection. In Section 2.3, the relationships between Chi-square statistic, mutual information and WMSD are discussed. In Section 3, the outstanding performance of the proposed method is numerically confirmed on both simulated and empirical datasets. Lastly, some conclusions of this study are given in Section 4. Some derivations and theoretical proofs are shown in the Appendix A and Appendix B.

## 2. Methodology

### 2.1. Weighted Mean Squared Deviation

As an general classification task, let ${\left(X_{i},Y_{i}\right)}_{1\leq i\leq n}$ be *n* independent identically distributed observations. For *i*-th observation, $X_{i}={\left(X_{i1},\cdots ,X_{ip}\right)}^{⊤}\in {\left\{0,1\right\}}^{p}$ is the associated *p*-dimensional binary feature, and $Y_{i}\in \left\{0,1\right\}$ is the corresponding binary class label. Denote all necessary parameters as follows, $P(Y_{i}=1)=\pi$, $P\left(X_{ij}=1|Y_{i}=1\right)=\theta_{1j}$, $P\left(X_{ij}=1|Y_{i}=0\right)=\theta_{0j}$, $P\left(X_{ij}Y_{i}=1\right)=\mu_{1j}=\pi \theta_{1j}$, $P\left(X_{ij}\left(1-Y_{i}\right)=1\right)=\mu_{0j}=\left(1-\pi \right)\theta_{0j}$ and $P\left(X_{ij}=1\right)=\theta_{j}=\pi \theta_{1j}+\left(1-\pi \right)\theta_{0j}$, for 1≤i≤n and 1≤j≤p. Under the model-free feature screening framework, we need to filter out the features that irrelevant (or independent) of class label, i.e., $\theta_{1j}=\theta_{0j}=\theta_{j}$. Intuitively, feature $X_{ij}$ is independent of $Y_{i}$, if and only if $\omega_{j}=\pi {\left(\theta_{1j}-\theta_{j}\right)}^{2}+\left(1-\pi \right){\left(\theta_{0j}-\theta_{j}\right)}^{2}=\pi \left(1-\pi \right){\left(\theta_{1j}-\theta_{0j}\right)}^{2}=0$. Note that, the probabilities of two classes are considered as weights in $\omega_{j}$. In contrast, *j*-th feature is relevant, if and only if $\omega_{j}\neq 0$. Then we define the true model as $T=\{j:\omega_{j}\neq 0,1\leq j\leq p\}$ with model size $\left|T\right|=d_{0}$ and the full model as F={1,⋯,p}.

Next, the Laplace smoothing method [18] is adopted for parameter estimation, to make all estimators bounded away from 0 and 1. The parameter estimators are denoted as $\hat{\pi }=\left(2+\sum_{i=1}^{n}Y_{i}\right)/\left(n+4\right)$, ${\hat{\mu }}_{1j}=\left(1+\sum_{i=1}^{n}Y_{i}X_{ij}\right)/\left(n+4\right)$ and ${\hat{\mu }}_{0j}=\left(1+\sum_{i=1}^{n}\left(1-Y_{i}\right)X_{ij}\right)/\left(n+4\right)$, for 1≤j≤p. It is easy to represent that ${\hat{\theta }}_{1j}={\hat{\mu }}_{1j}/\hat{\pi }$, ${\hat{\theta }}_{0j}={\hat{\mu }}_{0j}/\left(1-\hat{\pi }\right)$ and ${\hat{\theta }}_{j}={\hat{\mu }}_{1j}+{\hat{\mu }}_{0j}$, for 1≤j≤p. Then, a model-free feature screening statistic is constructed, called weighted mean squared deviation (WMSD), i.e.,

(1) $${\hat{\omega }}_{j}=\hat{\pi }\left(1-\hat{\pi }\right){\left({\hat{\theta }}_{1j}-{\hat{\theta }}_{0j}\right)}^{2},$$

which is an estimator of $\omega_{j}$. It is expected that, the features far away from independency should be selected. Intuitively, those features with larger ${\hat{\omega }}_{j}$ values are more likely to be relevant. In contrast, those with smaller ${\hat{\omega }}_{j}$ values are less likely. Consequently, an estimated model is defined as $\hat{M}=\left\{j:{\hat{\omega }}_{j}>c,j\in F\right\}$, where *c* is a positive critical value. The following theorem provides the asymptotic properties of the WMSD method under the assumption of ultrahigh dimension.

**Theorem** **1.** *Assume logp=o(n) and there exists a positive constant ϵ<1/3, such that ϵ≤π≤1−ϵ, $\epsilon \leq \theta_{kj}\leq 1-\epsilon$ for any k∈{0,1} and j∈F, and $|\theta_{1j}-\theta_{0j}|\geq \epsilon$ for j∈T. We have the following two results:*

(1) *$\max_{j\in F}\left|{\hat{\omega }}_{j}-\omega_{j}\right|=O_{P}\left(\sqrt{\log p/n}\right)$;*
(2) *there exists $0<c<\left(1-\epsilon \right)\epsilon^{3}$, such that $lim_{n\to \infty }P\left(\hat{M}=T\right)=1$.*

Note that, the conditions ϵ≤π≤1−ϵ, $\epsilon \leq \theta_{kj}\leq 1-\epsilon$ imply all parameters are bounded away from 0 and 1, and the condition $|\theta_{1j}-\theta_{0j}|\geq \epsilon$ implies $P\left(X_{ij}=1|Y_{i}=1\right)\neq P\left(X_{ij}=1|Y_{i}=0\right)$ for j∈T. Theorem 1 states that (1) ${\hat{\omega }}_{j}$ is a consistent estimator of $\omega_{j}$ and (2) $\hat{M}$ is a consistent estimator of T as long as the critical value *c* lies between 0 and $\left(1-\epsilon \right)\epsilon^{3}$, which is the strong screening consistency of WMSD. However, the lower bound ϵ is unknown in real applications. To this end, a practicable method is proposed in the next section. The proof of this theorem is left into Appendix A.

### 2.2. Feature Selection Via Pearson Correlation Coefficient

While the true model T can be theoretically selected by Theorem 1, it strongly depends on the critical value *c*. However, *c* is not given beforehand in empirical studies, and it always varies with the data. In order to solve this problem, the following strategy is developed for feature selection. Firstly, without loss of generality, it could be assumed that the features have been appropriately reordered such that ${\hat{\omega }}_{1}>{\hat{\omega }}_{2}>\cdots >{\hat{\omega }}_{p}$, then all candidate models can be given by $M=\{M_{\left(d\right)}:1\leq d\leq p\}$ with $M_{\left(d\right)}=\left\{1,\cdots ,d\right\}$ for 1≤d≤p, which is a finite set with a total of *p* nested candidate models. Thus, the original problem of determination for critical value *c* from (0,+∞) is converted into a model selection problem with respect to the model set M. Next, according to our best knowledge of text classification, the relatively large $\omega_{j}$s of irrelevant features approximatively follow a power-law distribution. Meanwhile, both $\omega_{j}$s of relevant features and relatively small $\omega_{j}$s of irrelevant features can not fit the power-law distribution well. The density function of power-law distribution can be represented as,

(2) $$p\left(x\right)=\frac{\alpha -1}{x_{0}}{\left(\frac{x}{x_{0}}\right)}^{-\alpha },$$

where the power parameter α>1 and the lower bound parameter $x_{0}>0$. A typical property of power-law distribution is that it obeys logp(x)=αlogx+C, i.e., it follows a straight line on a doubly logarithmic plot, where *C* is a constant dependent on parameters α and $x_{0}$. Therefore, a common way to probe for the power-law behavior is to construct the frequency distribution histogram of data, and plot the histogram on doubly logarithmic axes. If the doubly logarithmic histogram approximately falls on a straight line, the data can be considered to follow a power-law distribution [16]. This inspires us to use Pearson correlation coefficient of doubly logarithmic histogram of ${\hat{\omega }}_{j}$s to find an optimal model from M. The Pearson correlation coefficient of sequences ${\left\{\log j\right\}}_{1\leq j\leq m}$ and ${\left\{\log{\hat{\omega }}_{j}\right\}}_{d\leq j\leq d+m-1}$ can be represented as,

(3) $$r_{d}=\frac{m\sum_{j=1}^{m}\log j\log{\hat{\omega }}_{j+d-1}-\left(\sum_{j=1}^{m}\log j\right)\left(\sum_{j=1}^{m}\log{\hat{\omega }}_{j+d-1}\right)}{\sqrt{m\sum_{j=1}^{m}{\left(\log j\right)}^{2}-{\left(\sum_{j=1}^{m}\log j\right)}^{2}}\sqrt{m\sum_{j=1}^{m}{\left(\log{\hat{\omega }}_{j+d-1}\right)}^{2}-{\left(\sum_{j=1}^{m}\log{\hat{\omega }}_{j+d-1}\right)}^{2}}},$$

for 1≤d≤p−m+1, where *m* is the number of points when calculating Pearson correlation coefficient. Obviously, the absolute value of $r_{d}$ can be used to measure the approximate level of sequence ${\left\{{\hat{\omega }}_{j}\right\}}_{d\leq j\leq d+m-1}$ to power-law distribution. Thus, the best model is selected as $\hat{M}=M_{\left(\hat{d}\right)}$, with

(4) $$\hat{d}={\mathrm{argmax}}_{d_{min}\leq d\leq d_{max}}\left|r_{d}\right|-1,$$

where $d_{min}$ and $d_{max}$ are the smallest and largest true model sizes to be considered. In other words, if the sequence ${\left\{{\hat{\omega }}_{j}\right\}}_{\hat{d}+1\leq j\leq \hat{d}+m}$ fits the power-law distribution best over all candidate continuous subsequences of ${\left\{{\hat{\omega }}_{j}\right\}}_{1\leq j\leq p}$, then the features in model $\left\{\hat{d}+1\leq j\leq \hat{d}+m\right\}$ are more likely to be irrelevant and the features in model $\left\{1\leq j\leq \hat{d}\right\}$ are more likely to be relevant. As a result, the Pearson correlation coefficient method is adopted to determine the model size estimated by WMSD. In numerical studies, parameters *m*, $d_{min}$ and $d_{max}$ must be artificially given beforehand by empirical experience. The performance of numerical studies suggests that the feature selection method works quite well both on simulated and empirical data.

### 2.3. The Relationships between Chi-Square Statistic, Mutual Information and WMSD

As we know, Chi-square statistic and mutual information are two popularly used feature screening methods for discrete features. Next, the relationships between these two feature screening methods and WMSD will be investigated. According to the definitions of parameter estimators above, the Chi-square statistic can be represented as,

(5) $$\chi_{j}^{2}=\frac{n{\left\{n_{1j}\left(n-n_{1\cdot }-n_{\cdot j}+n_{1j}\right)-\left(n_{\cdot j}-n_{1j}\right)\left(n_{1\cdot }-n_{1j}\right)\right\}}^{2}}{n_{\cdot j}n_{1\cdot }\left(n-n_{\cdot j}\right)\left(n-n_{1\cdot }\right)}\approx n{\hat{\theta }}_{j}^{-1}{\left(1-{\hat{\theta }}_{j}\right)}^{-1}{\hat{\omega }}_{j},$$

where $n_{1\cdot }=\sum_{i=1}^{n}Y_{i}$, $n_{\cdot j}=\sum_{i=1}^{n}X_{ij}$, and $n_{1j}=\sum_{i=1}^{n}X_{ij}Y_{i}$ for 1≤j≤p. Formula (5) shows the relationship between Chi-square statistic and WMSD (see Appendix B.1 for detailed derivation). Thus, WMSD can be considered as a simplified version of Chi-square statistic.

In a similar way, the mutual information can be represented as,

(6) $$\begin{array}{ccc} MI_{j} & = & \frac{n_{1j}}{n}\log\frac{nn_{1j}}{n_{1\cdot }n_{\cdot j}}+\frac{n_{1\cdot }-n_{1j}}{n}\log\frac{n(n_{1\cdot }-n_{1j})}{n_{1\cdot }\left(n-n_{\cdot j}\right)}+\frac{n_{\cdot j}-n_{1j}}{n}\log\frac{n(n_{\cdot j}-n_{1j})}{n_{\cdot j}\left(n-n_{1\cdot }\right)}\\ & & +\frac{n-n_{1\cdot }-n_{\cdot j}+n_{1j}}{n}\log\frac{n(n-n_{1\cdot }-n_{\cdot j}+n_{1j})}{\left(n-n_{1\cdot }\right)\left(n-n_{\cdot j}\right)}\\ & \approx & n^{-1}\chi_{j}^{2}\approx {\hat{\theta }}_{j}^{-1}{\left(1-{\hat{\theta }}_{j}\right)}^{-1}{\hat{\omega }}_{j}, \end{array}$$

for 1≤j≤p, Formula (6) shows the relationship among mutual information, Chi-square statistic and WMSD (see Appendix B.2 for detailed derivation). Chi-square statistic and mutual information are asymptotic equivalent for feature screening of binary classification with binary features, if the sample size *n* is ignored.

**Remark** **1.** *From Formulas (5) and (6), compared to Chi-square statistic and mutual information, WMSD provides more opportunities to the features with probabilities (i.e., $\theta_{j}$) near 0.5. For an example, if n=100, ${\hat{\theta }}_{1}=0.2$, ${\hat{\theta }}_{2}=0.1$, $MI_{1}=0.2$ and $MI_{2}=0.3$, then $\chi_{1}^{2}\approx 20$, $\chi_{2}^{2}\approx 30$, ${\hat{\omega }}_{1}\approx 0.032$ and ${\hat{\omega }}_{2}\approx 0.027$. It is obviously that, $MI_{1}<MI_{2}$ and $\chi_{1}^{2}<\chi_{2}^{2}$, but ${\hat{\omega }}_{1}>{\hat{\omega }}_{2}$. This property is also confirmed in the following empirical study of Chinese text classification.*

## 3. Numerical Studies

### 3.1. Simulation Study

To evaluate the finite sample performance of WMSD feature screening method for binary classification with binary features, two standard feature selection methods are considered as competitors, i.e., Chi-square statistic (Chi2) and mutual information (MI). In addition, to investigate the robustness of the proposed method under different classifiers, two popular used classification methods are considered, i.e., naive Bayes (NB) and logistic regression (LR). To generate the simulated data, a multi-variate Bernoulli model [19] with both relevant and irrelevant binary features is considered. Moreover, different sample sizes of training set (i.e., n= 1000, 2000, 5000), different feature dimensions (i.e., p= 500, 1000), and different true model sizes (i.e., $d_{0}=$ 20, 50) are considered in parameter setup. For each fixed parameter setting, a total of 1000 simulation replications are conducted. For each simulated dataset, three feature screening methods are adopted, i.e., Chi2, MI and WMSD. Subsequently, the false positive rate (FPR), that is $\mathrm{FPR}=|T\backslash \hat{M}\left|/|T\right|$, of WMSD is calculated. In the same way, the false negative rate (FNR), that is $\mathrm{FNR}=|\left(F\backslash T\right)\cap \hat{M}\left|/|F\backslash T\right|$, of WMSD is also calculated. Average FPR and FNR values over 1000 replications are reported. Lastly, in order to evaluate the performance of classification, another 1000 independent observations as testing sample are generated for each replication. Then, the area under the receiver operating characteristic curve (AUC) is adopted to evaluate the out-of-sample prediction accuracy. The AUC values of NB and LR on three estimated models (separately selected by Chi2, MI and WMSD) are calculated on the testing sample and averaged over 1000 replications.

For the given simulation model and parameter setup, the simulated data is generated as follows. Firstly, generate the class label $Y_{i}\in \left\{0,1\right\}$ with probability $P(Y_{i}=1)=\pi =0.5$ for balanced case and π=0.8 for unbalanced case. Next, given $Y_{i}$, the *j*-th binary feature $X_{ij}$ is generated from a multi-variate Bernoulli model with probability $P\left(X_{ij}=1|Y_{i}=1\right)=\theta_{1j}=0.05\left\{j^{-0.2}p^{0.2}+I\left(1\leq j\leq 0.5d_{0}\right)j^{-0.5}d_{0}^{0.5}\right\}$ and $P\left(X_{ij}=1|Y_{i}=0\right)=\theta_{0j}=0.05\left\{j^{-0.2}p^{0.2}+I\left(0.5d_{0}+1\leq j\leq d_{0}\right)j^{-0.5}d_{0}^{0.5}\right\}$ for j∈{1,⋯,p}, where I(·) is the indicator function. Note that, without loss of generality, we set $T=\{1,\cdots ,d_{0}\}$, that is, the first $d_{0}$ features are relevant. Moreover, in this simulation, the parameters in Formulas (3) and (4) are set to be m=100, $d_{min}=10$ and $d_{max}=100$.

The detailed simulation results are given in Table 1. In balanced case (i.e., π=0.5), the following results could be obtained. First, if both *p* and *n* are fixed, a larger true model size $d_{0}$ leads to a larger AUC. Because the more relevant features are involved, the better we can predict. Second, if both $d_{0}$ and *n* are fixed, a larger feature dimension *p* leads to worse performance in terms of AUC. This is reasonable because the larger feature dimension leads to more challenge for feature selection and then a worse prediction. Third, if both *p* and $d_{0}$ are fixed, a larger sample size *n* leads to a larger AUC and a smaller FPR. This is expected because the larger sample size leads to a more accurate estimator and then a better prediction. Forth, in almost all parameter settings, the AUC values of WMSD are larger than that of Chi2 and MI, which states that WMSD performs better than the other two methods on the simulated data. Last, for all parameter settings, the FNR values are relatively small, which indicates that WMSD can filter out most irrelevant features. The results of unbalanced case (i.e., π=0.8) are similar to that of balanced case. For any parameter setting, FPR values are larger than that of balanced case, which implies that feature selection is harder in unbalanced case.

### 3.2. An Application in Chinese Text Classification

The dataset is downloaded from CNKI (www.cnki.net), which is one of the largest academic literature platform in China. It contains n= 14,473 abstracts of articles published in CSSCI (Chinese Social Sciences Citation Index) journals of economics and management fields in 2018. The abstracts are composed of p=2385 Chinese words (ignored the words with frequencies less than 10). Our purpose is to classify the articles into different fields (economics or management) according to their abstracts, and select a small number of feature words which are helpful for classification. Economics or management is considered as class 1 (i.e., $Y_{i}=1$) and the other is considered as class 0 (i.e., $Y_{i}=0$), respectively. In summary, there are 8570 abstracts from economics and 5903 from management. To this end, naive Bayes and logistic regression are both considered as standard classification methods. Then, Chi-square statistic, mutual information and WMSD are considered as feature screening methods and the performances of them are compared based on the two classification methods. It is noted that, the results of these feature selection methods are invariable when class 1 and class 0 are exchanged.

**Figure 1:**
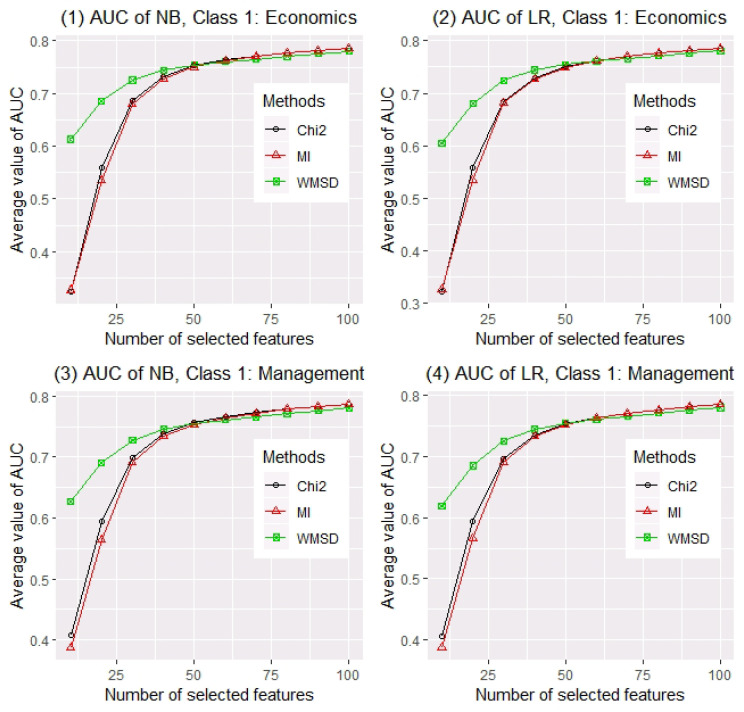
Averaged AUC values of NB and LR on three models ranked by Chi2, MI and WMSD with different model sizes (from 10 to 100 by 10), when economics and management are considered as class 1, over 200 replications.

Next, we sample 10,000 abstracts as the training set and the others as the testing set randomly. For comparison of feature screening methods, different numbers of selected words *d* (from 10 to 100 by 10) are considered. The AUC values of two classification methods with different numbers of selected words are calculated for evaluating feature screening methods. For each setting, a total of 200 random replications are conducted. The averaged AUC values of two classifiers (i.e., NB and LR) over 200 replications for three feature screening methods (i.e., Chi2, MI and WMSD) with different number of selected words, when economics and management are considered as class 1 respectively, are reported in Figure 1. Panel (1) of Figure 1 shows that when naive Bayes classifier is applied and economics is considered as class 1, AUC values based on three estimated models (separately selected by Chi2, MI and WMSD) increase as *d* becomes larger. Obviously, WMSD far outperforms other methods when d<50, and they perform similarly when d≥50. Panel (2) shows a similar result as panel (1) when logistic regression is applied. Panels (3) and (4) of Figure 1 show that, WMSD also far outperforms Chi2 and MI when d<50, if the classes are exchanged.

**Figure 2:**
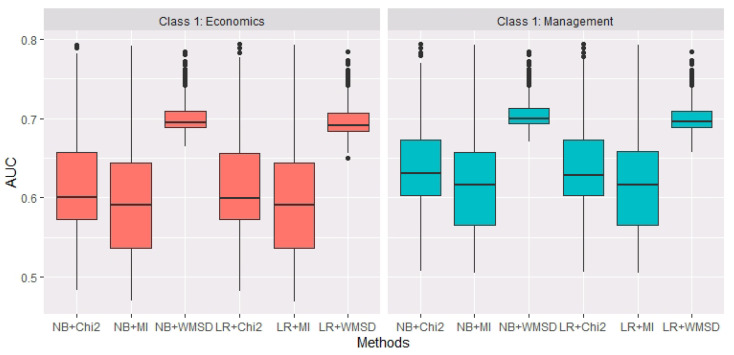
The boxplots of AUC values of NB and LR based on three estimated models by Chi2, MI and WMSD, when economics and management are considered as class 1, over 200 replications.

Furthermore, the Pearson correlation coefficient method is used to determine the estimated model size of WMSD. To calculate $\hat{d}$, the parameters in Formulas (3) and (4) are set to be m=100, $d_{min}=20$ and $d_{max}=100$. The averaged $\hat{d}$ is 25.86 over 200 replications. In each replication, for the same $\hat{d}$, AUC values of NB and LR based on three estimated models by Chi2, MI, WMSD are calculated separately. Figure 2 shows the boxplots of AUC for six situations (i.e., NB+Chi2, NB+MI, NB+WMSD, LR+Chi2, LR+MI and LR+WMSD) over 200 replications. It could be observed that, when the estimated model size is relatively small (actually, averaged $\hat{d}$ is 25.86), WMSD performs more accurate and robust than Chi2 and MI in terms of AUC, whether economics or management is considered as class 1.

Lastly, the probabilities of top 10 words ranked by three feature screening methods are also calculated separately, based on all *n* = 14,473 abstracts. It can be seen from Table 2 that the probabilities of top 10 words ranked by WMSD are larger than that of other two methods. It states that WMSD provides more opportunities to high frequency words (with probabilities near 0.5). Because the word frequencies of almost all words are less than 0.5, the word frequencies of high frequency words are closer to 0.5. It validates the property of WMSD mentioned in Section 2.3.

## 4. Conclusions

In this study, a novel model-free feature screening method called weighted mean squared deviation is proposed especially for ultrahigh dimensional binary features of binary classification, which is a measure of dependence between each feature and the class label. WMSD can be considered as a simplified version of Chi-square statistic and mutual information, which can provide more opportunities to the features with probabilities near 0.5. Furthermore, the strong screening consistency of WMSD is investigated theoretically, the number of features is determined by a Pearson correlation coefficient method practically, and the performance of WMSD is numerically confirmed both on simulated data and an real example of Chinese text classification. Three potential directions are also proposed for future studies. First, for multi-class classification with categorical features, the corresponding WMSD statistics need to be theoretically and numerically investigated. Second, the feature selection method via the Pearson correlation coefficient has not been theoretically verified, which is an important problem to be solved. Last, in order to further confirm the outstanding performance of WMSD in empirical research, it may make sense to investigate specifically the observations for which other methods give a probability near 0.5 (i.e., it is hard to predict their class labels) in future studies.

## Figures and Tables

**Table 1:** Results of simulation study. The averaged area under the receiver operating characteristic curve (AUC) values of naive Bayes (NB) and logistic regression (LR) based on three estimated models (Chi-square statistic (Chi2), mutual information (MI) and weighted mean squared deviation (WMSD)) are reported, and the averaged false positive rate (FPR) and false negative rate (FNR) values of WMSD are also reported, over 1000 replications.

			AUC of NB			AUC of LR				
$d_{0}$	*p*	*n*	Chi2	MI	WMSD	Chi2	MI	WMSD	FPR	FNR
π=0.5										
20	500	1000	0.7238	0.7233	0.7318	0.6966	0.6960	0.7033	0.4188	0.0001
		2000	0.7610	0.7609	0.7625	0.7411	0.7411	0.7428	0.1930	0.0000
		5000	0.7778	0.7778	0.7779	0.7673	0.7673	0.7676	0.0108	0.0013
	1000	1000	0.7145	0.7135	0.7303	0.6849	0.6839	0.7007	0.4014	0.0001
		2000	0.7545	0.7543	0.7591	0.7335	0.7332	0.7399	0.1599	0.0001
		5000	0.7693	0.7693	0.7697	0.7584	0.7584	0.7592	0.0024	0.0010
50	500	1000	0.8936	0.8935	0.8973	0.8463	0.8460	0.8499	0.2976	0.0008
		2000	0.9102	0.9102	0.9110	0.8837	0.8837	0.8850	0.1058	0.0001
		5000	0.9165	0.9165	0.9165	0.8998	0.8998	0.8998	0.0096	0.0005
	1000	1000	0.8789	0.8787	0.8851	0.8239	0.8233	0.8313	0.3408	0.0004
		2000	0.9014	0.9013	0.9031	0.8717	0.8716	0.8748	0.1106	0.0001
		5000	0.9097	0.9097	0.9098	0.8921	0.8921	0.8923	0.0017	0.0007
π=0.8										
20	500	1000	0.6372	0.6502	0.6883	0.6422	0.6545	0.6905	0.4796	0.0007
		2000	0.7206	0.7237	0.7303	0.7203	0.7239	0.7307	0.3413	0.0001
		5000	0.7692	0.7692	0.7696	0.7658	0.7659	0.7664	0.0706	0.0001
	1000	1000	0.6171	0.6329	0.6908	0.6268	0.6405	0.6936	0.4833	0.0007
		2000	0.7183	0.7210	0.7328	0.7190	0.7216	0.7330	0.3214	0.0001
		5000	0.7642	0.7640	0.7658	0.7614	0.7613	0.7627	0.0406	0.0002
50	500	1000	0.8636	0.8665	0.8746	0.8537	0.8542	0.8594	0.4739	0.0017
		2000	0.9018	0.9022	0.9043	0.8930	0.8923	0.8935	0.2115	0.0005
		5000	0.9149	0.9149	0.9150	0.9107	0.9107	0.9107	0.0442	0.0000
	1000	1000	0.8428	0.8468	0.8583	0.8326	0.8337	0.8425	0.5433	0.0008
		2000	0.8894	0.8899	0.8943	0.8790	0.8783	0.8821	0.2291	0.0004
		5000	0.9075	0.9074	0.9079	0.9028	0.9027	0.9034	0.0295	0.0001

**Table 2:** The probabilities of top 10 words ranked by three feature screening methods, Chi2, MI and WMSD.

Methods	Probabilities of Top 10 Words									
Chi2	0.285	0.034	0.133	0.029	0.043	0.047	0.012	0.014	0.022	0.017
MI	0.285	0.133	0.034	0.029	0.022	0.043	0.026	0.019	0.012	0.047
WMSD	0.285	0.133	0.541	0.211	0.223	0.203	0.034	0.235	0.047	0.043

## Appendix A. Proof of Theorem 1

According to the definitions of $\hat{\pi }$, ${\hat{\mu }}_{1j}$ and ${\hat{\mu }}_{0j}$, we know that they all lie between ${\left(n+4\right)}^{-1}$ and $1-{\left(n+4\right)}^{-1}$. In addition, by the conditions of Theorem 1, it is also known that π, $\theta_{1j}$ and $\theta_{0j}$ are all bounded away from 0 and 1 for j∈F. Then $\mu_{1j}$ and $\mu_{0j}$ are also bounded away from 0 and 1 for j∈F. By the conclusions of Lemma 1 in [12], for any ε>0 and sufficiently large *n*, we have $P(|\hat{\pi }-\pi \left|>\varepsilon \right)\leq 2\exp\left(-2n\varepsilon^{2}\right)$, and $P(|{\hat{\mu }}_{kj}-\mu_{kj}\left|>\varepsilon \right)\leq 2\exp\left(-2n\varepsilon^{2}\right)$, for k=0,1 and 1≤j≤p. In addition, $\omega_{j}$ and ${\hat{\omega }}_{j}$ can also be rewritten as

$$\omega_{j}=\pi \left(1-\pi \right){\left\{\pi^{-1}\mu_{1j}-{\left(1-\pi \right)}^{-1}\mu_{0j}\right\}}^{2},$$

$${\hat{\omega }}_{j}=\hat{\pi }\left(1-\hat{\pi }\right){\left\{{\hat{\pi }}^{-1}{\hat{\mu }}_{1j}-{\left(1-\hat{\pi }\right)}^{-1}{\hat{\mu }}_{0j}\right\}}^{2}.$$

Then, by the conclusion of Lemma 2 in [12], for any ε>0, we have $P(|{\hat{\omega }}_{j}-\omega_{j}\left|>\varepsilon \right)\leq C_{1}\exp\left(-C_{2}n\varepsilon^{2}\right)$, where $C_{1}$ and $C_{2}$ are some positive constants. Next, by Bonferroni’s inequality [20],

$$P\left(\max_{j\in F}\left|{\hat{\omega }}_{j}-\omega_{j}\right|>\sqrt{2/C_{2}}\sqrt{\log p/n}\right)\leq \sum_{j=1}^{p}P\left(|{\hat{\omega }}_{j}-\omega_{j}|>\sqrt{2/C_{2}}\sqrt{\log p/n}\right)$$

$$\leq pC_{1}\exp\left\{-C_{2}\left(2/C_{2}\right)\log p\right\}=C_{1}\exp\left(-\log p\right)\to 0.$$

Consequently, we know that $\max_{j\in F}\left|{\hat{\omega }}_{j}-\omega_{j}\right|=O_{P}\left(\sqrt{\log p/n}\right)$.

By the condition of Theorem 1, ϵ≤π≤1−ϵ and $|\theta_{1j}-\theta_{0j}|\geq \epsilon$ for j∈T, we have $\omega_{j}=\pi \left(1-\pi \right){\left(\theta_{1j}-\theta_{0j}\right)}^{2}\geq \left(1-\epsilon \right)\epsilon^{3}$ for j∈T and $\omega_{j}=0$ for j∉T. For $0<c<\left(1-\epsilon \right)\epsilon^{3}$ and logp=o(n), we have

$$\begin{array}{ccc} P(\hat{M}=T) & = & P\left(\min_{j\in T}{\hat{\omega }}_{j}>c,\max_{j\notin T}{\hat{\omega }}_{j}<c\right)\\ & \geq & P\left(\min_{j\in T}{\hat{\omega }}_{j}>c\right)+P\left(\max_{j\notin T}{\hat{\omega }}_{j}<c\right)-1\\ & \geq & P\left(\max_{j\in T}\left|{\hat{\omega }}_{j}-\omega_{j}\right|<\left(1-\epsilon \right)\epsilon^{3}-c\right)+P\left(\max_{j\notin T}\left|{\hat{\omega }}_{j}-\omega_{j}\right|<c\right)-1. \end{array}$$

Hence $P(\hat{M}=T)\to 1$ as n→∞. The proof is completed.

## Appendix B. Some Necessary Derivations

### Appendix B.1. Derivation of the Relationship between Chi-Square Statistic and WMSD

Denote $n_{1\cdot }=\sum_{i=1}^{n}Y_{i}$, $n_{\cdot j}=\sum_{i=1}^{n}X_{ij}$, and $n_{1j}=\sum_{i=1}^{n}X_{ij}Y_{i}$. According to the definitions of estimators, we have $\hat{\pi }\approx n_{1\cdot }/n$, ${\hat{\theta }}_{j}\approx n_{\cdot j}/n$, ${\hat{\theta }}_{1j}\approx n_{1j}/n_{1\cdot }$ and ${\hat{\theta }}_{0j}\approx \left(n_{\cdot j}-n_{1j}\right)/\left(n-n_{1\cdot }\right)$. Then we have

$$\begin{array}{ccc} \chi_{j}^{2} & = & \frac{n{\left\{n_{1j}\left(n-n_{1\cdot }-n_{\cdot j}+n_{1j}\right)-\left(n_{\cdot j}-n_{1j}\right)\left(n_{1\cdot }-n_{1j}\right)\right\}}^{2}}{n_{\cdot j}n_{1\cdot }\left(n-n_{\cdot j}\right)\left(n-n_{1\cdot }\right)}\\ & \approx & n{\hat{\theta }}_{j}^{-1}{\left(1-{\hat{\theta }}_{j}\right)}^{-1}\hat{\pi }\left(1-\hat{\pi }\right){\left({\hat{\theta }}_{1j}-{\hat{\theta }}_{0j}\right)}^{2}\\ & = & n{\hat{\theta }}_{j}^{-1}{\left(1-{\hat{\theta }}_{j}\right)}^{-1}{\hat{\omega }}_{j}. \end{array}$$

It is the relationship between Chi-square statistic and WMSD.

### Appendix B.2. Derivation of the Relationship between Mutual Information and WMSD

Based on the notations used in Appendix B.1 and according to the Taylor’s theorem, we have

$$\begin{array}{ccc} MI_{j} & = & \frac{n_{1j}}{n}\log\frac{nn_{1j}}{n_{1\cdot }n_{\cdot j}}+\frac{n_{1\cdot }-n_{1j}}{n}\log\frac{n(n_{1\cdot }-n_{1j})}{n_{1\cdot }\left(n-n_{\cdot j}\right)}+\frac{n_{\cdot j}-n_{1j}}{n}\log\frac{n(n_{\cdot j}-n_{1j})}{n_{\cdot j}\left(n-n_{1\cdot }\right)}\\ & & +\frac{n-n_{1\cdot }-n_{\cdot j}+n_{1j}}{n}\log\frac{n(n-n_{1\cdot }-n_{\cdot j}+n_{1j})}{\left(n-n_{1\cdot }\right)\left(n-n_{\cdot j}\right)}\\ & = & \hat{\pi }\left[{\hat{\theta }}_{1j}\log\left({\hat{\theta }}_{1j}/{\hat{\theta }}_{j}\right)+\left(1-{\hat{\theta }}_{1j}\right)\log\left\{\left(1-{\hat{\theta }}_{1j}\right)/\left(1-{\hat{\theta }}_{j}\right)\right\}\right]\\ & & +\left(1-\hat{\pi }\right)\left[{\hat{\theta }}_{0j}\log\left({\hat{\theta }}_{0j}/{\hat{\theta }}_{j}\right)+\left(1-{\hat{\theta }}_{0j}\right)\log\left\{\left(1-{\hat{\theta }}_{0j}\right)/\left(1-{\hat{\theta }}_{j}\right)\right\}\right]\\ & \approx & \hat{\pi }\left[{\hat{\theta }}_{1j}\left({\hat{\theta }}_{1j}/{\hat{\theta }}_{j}-1\right)+\left(1-{\hat{\theta }}_{1j}\right)\left\{\left(1-{\hat{\theta }}_{1j}\right)/\left(1-{\hat{\theta }}_{j}\right)-1\right\}\right]\\ & & +\left(1-\hat{\pi }\right)\left[{\hat{\theta }}_{0j}\left({\hat{\theta }}_{0j}/{\hat{\theta }}_{j}-1\right)+\left(1-{\hat{\theta }}_{0j}\right)\left\{\left(1-{\hat{\theta }}_{0j}\right)/\left(1-{\hat{\theta }}_{j}\right)-1\right\}\right]\\ & = & \hat{\pi }\left(1-\hat{\pi }\right){\left({\hat{\mu }}_{1j}+{\hat{\mu }}_{0j}\right)}^{-1}{\left(1-{\hat{\mu }}_{1j}-{\hat{\mu }}_{0j}\right)}^{-1}{\left\{{\hat{\pi }}^{-1}{\hat{\mu }}_{1j}-{\left(1-\hat{\pi }\right)}^{-1}{\hat{\mu }}_{0j}\right\}}^{2}\\ & = & n^{-1}\chi_{j}^{2}. \end{array}$$

As a result, we know that $MI_{j}\approx {\hat{\theta }}_{j}^{-1}{\left(1-{\hat{\theta }}_{j}\right)}^{-1}{\hat{\omega }}_{j}$. It is the relationship between mutual information and WMSD.
